# Supplementary material for: Opportunistic screening for type 2 diabetes in community pharmacies. Results from a region-wide experience in Italy
Source: PLoS One. 2020 Mar 18;15(3):e0229842. doi: 10.1371/journal.pone.0229842 (PMC7080237; doi:10.1371/journal.pone.0229842)
Supplement: Attachment — Submitted filename: Response to reviewers.docx [file pone.0229842.s001.docx]

**RESPONSE TO ACADEMIC EDITOR AND REVIEWERS’ COMMENTS**

**Dear Academic Editor,**

**Please, find below highlighted in bold our response to your comments and those of the reviewers**

PONE-D-19-35176
Opportunistic screening for type 2 diabetes in community pharmacies.  Results from a region-wide experience in Italy.
PLOS ONE

If you would like to make changes to your financial disclosure, please include your updated statement in your cover letter.

**We updated our disclosure in the cover letter.**

To enhance the reproducibility of your results, we recommend that if applicable you deposit your laboratory protocols in protocols.io, where a protocol can be assigned its own identifier (DOI) such that it can be cited independently in the future.

**We have uploaded our minimal data set to zenodo.org website and reported the DOI in the revised cover letter, according to your instructions.**

Please include the following items when submitting your revised manuscript:

- A rebuttal letter that responds to each point raised by the academic editor and reviewer(s). This letter should be uploaded as separate file and labeled 'Response to Reviewers'.

**A rebuttal letter has been uploaded.**

- A marked-up copy of your manuscript that highlights changes made to the original version. This file should be uploaded as separate file and labeled 'Revised Manuscript with Track Changes'.

**A marked copy has been uploaded.**

- An unmarked version of your revised paper without tracked changes. This file should be uploaded as separate file and labeled 'Manuscript'.

**An unmarked copy has been uploaded.**

Journal Requirements:

When submitting your revision, we need you to address these additional requirements.

Please ensure that your manuscript meets PLOS ONE's style requirements, including those for file naming. The PLOS ONE style templates can be found at http://www.journals.plos.org/plosone/s/file?id=wjVg/PLOSOne_formatting_sample_main_body.pdf and <http://www.journals.plos.org/plosone/s/file?id=ba62/PLOSOne_formatting_sample_title_authors_affiliations.pdf>

**We double checked the manuscript to ensure that it meets the PLOS ONE style requirements.**

2. Thank you for including your ethics statement:  "Ethical Committee Approval Protocol n°46480/13".   Please amend your current ethics statement to include the full name of the ethics committee/institutional review board(s) that approved your specific study.

Once you have amended this/these statement(s) in the Methods section of the manuscript, please add the same text to the “Ethics Statement” field of the submission form (via “Edit Submission”).

**We amended the ethic statement.**

3. We note that you have indicated that data from this study are available upon request. PLOS only allows data to be available upon request if there are legal or ethical restrictions on sharing data publicly

**We have uploaded our minimal data set to zenodo.org website and reported the DOI in the revised cover letter, according to your instructions.**

We will update your Data Availability statement on your behalf to reflect the information you provide.

4. Thank you for providing the following Funding Statement:

"This work was supported by a research grant from the Italian Ministry of Health (www.salute.gov.it) within the Programma CCM 2015: “La farmacia dei servizi per il controllo delle patologie croniche: sperimentazione e trasferimento di un modello di intervento di prevenzione sul diabete di tipo 2”, and an unconditional grant from Federfarma Piemonte ([www.federfarma.it](http://www.federfarma.it)).
The funders had no role in study design, data collection and analysis, decision to publish, or preparation of the manuscript."

We note that one or more of the authors is affiliated with the funding organization "Federfarma Piemont", indicating the funder may have had some role in the design, data collection, analysis or preparation of your manuscript for publication; in other words, the funder played an indirect role through the participation of the co-authors.

**We believe that is necessary to clarify that Federfarma is a syndicate (representative of pharmacists), not a company with commercial purposes. Furthermore, Dr Massimo Mana took part in the study mainly as chief of ATF Informatics, which has provided the specific database for data analysis. To avoid misunderstandings, we preferred to modify the affiliation of Dr Mana accordingly. His affiliation in Federfarma was declared because he is the Regional President, and we meant it as thanks to all the affiliated pharmacies that participated in the study; this role is not relevant for our research. In fact, Federfarma only provided financial support in the form of research materials (such as questionnaires), and did not have any additional role in the management of the study.**

If the funding organization did not play a role in the study design, data collection and analysis, decision to publish, or preparation of the manuscript and only provided financial support in the form of authors' salaries and/or research materials, please review your statements relating to the author contributions, and ensure you have specifically and accurately indicated the role(s) that these authors had in your study in the Author Contributions section of the online submission form. Please make any necessary amendments directly within this section of the online submission form.  Please also update your Funding Statement to include the following statement: “The funder provided support in the form of salaries for authors [insert relevant initials], but did not have any additional role in the study design, data collection and analysis, decision to publish, or preparation of the manuscript. The specific roles of these authors are articulated in the ‘author contributions’ section.”

If the funding organization did have an additional role, please state and explain that role within your Funding Statement.

Please also provide an updated Competing Interests Statement declaring this commercial affiliation along with any other relevant declarations relating to employment, consultancy, patents, products in development, or marketed products, etc.

Within your Competing Interests Statement, please confirm that this commercial affiliation does not alter your adherence to all PLOS ONE policies on sharing data and materials by including the following statement: "This does not alter our adherence to  PLOS ONE policies on sharing data and materials.” (as detailed online in our guide for authors <http://journals.plos.org/plosone/s/competing-interests>). If this adherence statement is not accurate and  there are restrictions on sharing of data and/or materials, please state these. Please note that we cannot proceed with consideration of your article until this information has been declared.

Please know it is PLOS ONE policy for corresponding authors to declare, on behalf of all authors, all potential competing interests for the purposes of transparency. PLOS defines a competing interest as anything that interferes with, or could reasonably be perceived as interfering with, the full and objective presentation, peer review, editorial decision-making, or publication of research or non-research articles submitted to one of the journals. Competing interests can be financial or non-financial, professional, or personal. Competing interests can arise in relationship to an organization or another person. Please follow this link to our website for more details on competing interests: <http://journals.plos.org/plosone/s/competing-interests>


Reviewers' comments:

Reviewer's Responses to Questions

**Comments to the Author**

1. Is the manuscript technically sound, and do the data support the conclusions?

The manuscript must describe a technically sound piece of scientific research with data that supports the conclusions. Experiments must have been conducted rigorously, with appropriate controls, replication, and sample sizes. The conclusions must be drawn appropriately based on the data presented.

Reviewer #1: Partly

Reviewer #2: Yes

 2. Has the statistical analysis been performed appropriately and rigorously?

Reviewer #1: Yes

Reviewer #2: Yes

3. Have the authors made all data underlying the findings in their manuscript fully available?

The [PLOS Data policy](http://www.plosone.org/static/policies.action#sharing) requires authors to make all data underlying the findings described in their manuscript fully available without restriction, with rare exception (please refer to the Data Availability Statement in the manuscript PDF file). The data should be provided as part of the manuscript or its supporting information, or deposited to a public repository. For example, in addition to summary statistics, the data points behind means, medians and variance measures should be available. If there are restrictions on publicly sharing data—e.g. participant privacy or use of data from a third party—those must be specified.

Reviewer #1: Yes

Reviewer #2: Yes

4. Is the manuscript presented in an intelligible fashion and written in standard English?

PLOS ONE does not copyedit accepted manuscripts, so the language in submitted articles must be clear, correct, and unambiguous. Any typographical or grammatical errors should be corrected at revision, so please note any specific errors here.

Reviewer #1: Yes

Reviewer #2: Yes

5. Review Comments to the Author

Please use the space provided to explain your answers to the questions above. You may also include additional comments for the author, including concerns about dual publication, research ethics, or publication ethics. (Please upload your review as an attachment if it exceeds 20,000 characters)

**Reviewer #1:** The study by Roberto Gnavi et al. aims to evaluate an opportunistic screening strategy based on FINDRISC score for the identification of individuals with type 2 diabetes in Italy.
I would like to applaud the authors impressive efforts of engaging and retain such a large number of pharmacies to voluntarily contribute to the study.

We thank the reviewer fir his/her appreciation of our work.

The manuscript is well written and data clearly presented. I have 2 major concerns:

1. The authors mention that no population screening is recommended in Italy. In contrast, North America has several screening guideline recommendations (American Association of Clinical Endocrinologists, American Diabetes Association, Canadian Task Force on Preventive Health Care, and U.S. Preventive Services Task Force) each with their individual set of parameters. What was the authors rationale to use FINDRISC score?

**Our statement that population screening is not recommended comes from what reported by some European agencies such as the British National Institute for health and Care Excellence (NICE), summarized in the reference 13, and refers to systematic population-based screening. Opportunistic screening is considered a valid alternative to structured population screening. For these purposes, FINDRISC is widely used as a first level screening test, as it has shown its efficacy in predicting the probability of developing type 2 diabetes in the following 10 years. As we reported in the introduction of our paper, FINDRISC has also been validated in Italy. Given its efficacy and its ease of use, we considered it as an appropriate tool to be used in the pharmacy setting.**

**At the same time, we agree with the reviewer that recommendations are not homogenous among countries and scientific societies. Therefore, to underline that there is no clear agreement regarding recommendations, the sentence in the introduction has been rewritten in this way:**

***“ …This implies that, even if the efficacy of systematic population-based screening is still under debate and not recommended by some health institutions…”***

Was there any cost effectiveness analysis performed?

**We are aware that our study would benefit of a cost effectiveness analysis. Indeed, we are considering to extend our analysis by adding an in-depth analysis in this regard.**

2. The authors mention in the Results section that “pharmacies were randomized to the two arms of the project and 443 were assigned to opportunistic screening”. It is unclear who are the subjects in the control group. The way it is initially described as a cross sectional study and it is not clear at what point randomization occurred. If there was a control group the authors need to describe the results for this group of patients: how many patients were diagnosed with DM in this group? If there was no control group the authors should present data on the pharmacies that were not assigned to the opportunistic screening and then compare it with the intervention group. In this way the validity of the FINDRISC score could be tested in this highly selected group.

**We indeed randomised to the two arms of the intervention (the second arm being the evaluation of treatment adherence among patients, whose results are not reported in this paper). We understand that the term “randomization” can be interpreted ambiguously. The sentence was then rewritten as “…. 443 pharmacies were assigned to opportunistic screening…”.**

**A randomization with a control group was not possible as it was not possible to enrol a control group similar to the screened group. This would have implied to collect individual data (at least sex and age) of customers at potential risk of developing diabetes (overweight and age 45 or more) entering pharmacies not participating to the intervention, without doing any intervention, and following them up for one year to monitor the onset of diabetes. We considered this, first, as an unethical option, secondly as almost impossible to achieve.**

**For this reason we reported: “….. Since the programme was launched simultaneously throughout the region, we could not introduce a pharmacy-based control group. Therefore, we used historical data to estimate the incidence of diabetes in the absence of screening and calculate the additional number of newly diagnosed cases due to the screening process…” (page 6, lines 140-143).**

**Reviewer #2**: Gnavi et al, used the FRINDISC questionnaire in community pharmacies to identify patients at risk for diabetes mellitus type 2 (DMT2) in overweight, diabetes free customers aged >45 years. Excluding the number of cases expected during one year follow-up, per 117 interviews one new case of DMT2 was identified. This is an interesting and important study that is easily implemented in daily practice,

**We thank the reviewer fir his/her appreciation of our work**

Comments:

Results:
Page 9: age is part of the FRINDISC score, so it is evident that higher age is associated with a higher score. Please omit age in this sentence.

**We omitted age from the sentence as suggested by the reviewer.**

Table 1:
First line: either provide the numbers for men or women.

**We agree that reporting the number of both men and women is a kind of repetition; however, considering that it is only one single line, for greater clarity to readers, we would prefer to maintain both genders.**

High pressure medication should be replaced by: anti-hypertensive medication.
High glicemic levels, should be replaced by “glycemic”. Please define hyperglycemia in the legenda of this Table.
Define: low physical activity and low vegetable consumption in the legend.
What do the authors mean by “familiarity”?

**We added to table 1 a legenda that reports the FINDRISC definitions of the items.**

Table 2
“Glicemia” should be replaced by “glycemic”.

**Done.**
